# Weight Change After Subthalamic Nucleus Deep Brain Stimulation in Patients With Isolated Dystonia

**DOI:** 10.3389/fneur.2021.632913

**Published:** 2021-02-24

**Authors:** Weibin He, Hongxia Li, Yijie Lai, Yunhao Wu, Yiwen Wu, Adolfo Ramirez-Zamora, Wei Yi, Chencheng Zhang

**Affiliations:** ^1^Department of Neurosurgery, Renmin Hospital, Wuhan University, Wuhan, China; ^2^Department of Neurology, Ruijin Hospital, Shanghai Jiao Tong University School of Medicine, Shanghai, China; ^3^Department of Functional Neurosurgery, Ruijin Hospital, Shanghai Jiao Tong University School of Medicine, Shanghai, China; ^4^Fixel Center for Neurological Diseases, University of Florida, Gainesville, FL, United States

**Keywords:** isolated dystonia, subthalamic nucleus, deep brain stimulation, Burke-Fahn-Marsden dystonia rating scale, weight gain

## Abstract

**Purpose:** Deep brain stimulation of the subthalamic nucleus (STN-DBS) is an effective treatment method for advanced Parkinson's disease (PD) and isolated dystonia and provides marked improvement of major motor symptoms. In addition, non-motor effects have been reported including weight gain (WG) in patients with PD after STN-DBS. However, it is still unclear whether patients with isolated dystonia also experience WG.

**Methods:** Data from 47 patients with isolated dystonia who underwent bilateral STN-DBS surgery between October 2012 and June 2019 were retrospectively collected. The severity of dystonia was assessed via the Burke–Fahn–Marsden Dystonia Rating Scale (BFMDRS). Changes in the body mass index (BMI) and BFMDRS score were analyzed using paired Student's *t*-tests. Regression analysis was performed to identify factors that affected the BMI after surgery.

**Results:** Postoperative WG was observed in 78.7% of patients. The percentage of overweight and obese patients increased from 25.5% (before STN-DBS) to 48.9% (at the last follow-up). The mean BMI and mean percentage change in BMI increased by 1.32 ± 1.83 kg/m^2^ (*P* < 0.001) and 6.28 ± 8.34%, respectively. BMI increased more in female than in male patients. At the last follow-up, BFMDRS movement and disability scores improved by 69.76 ± 33.23% and 65.66 ± 31.41%, respectively (both *P* < 0.001). The final regression model analysis revealed that sex and preoperative BMI alone were independently associated with BMI change (*P* < 0.05).

**Conclusions:** STN-DBS is associated with postoperative WG with patients with isolated dystonia. WG is more prominent in female patients and is associated with preoperative weight but not with the efficacy of STN-DBS on motor symptoms.

## Introduction

In recent decades, deep brain stimulation (DBS) has been applied to treat many disorders, such as advanced stages of Parkinson's disease (PD), intractable essential tremor, complicated segmental and generalized dystonia, and neuropsychiatric diseases (1, 2). DBS may improve patients' quality of life and result in desired therapeutic effects such as reduced tremor, dyskinesia, and stiffness in patients with PD (3, 4). However, DBS can also cause adverse events (5) and side effects (e.g., cognitive and emotional changes) (6–8) in some patients.

Interestingly, many researchers have reported that weight gain (WG) is common in patients with PD after DBS of the subthalamic nucleus (STN-DBS) (9). This is concerning because WG may lead to additional health complications such as obesity and diabetes (6). Some potential mechanisms underlying WG in patients with PD after STN-DBS treatment have been suggested, including the improvement of resting tremor and dyskinesias, reduction in energy expenditure (EE), changes in eating behavior and food intake, perturbations of homeostatic control, changes in hormone and neurotransmitter systems, and improvement in motor function; however, the exact mechanism underlying WG remains unknown (9).

Dystonia is characterized by sustained and involuntary muscular contractions, which result from dysfunction of the cortico-striato-thalamo-cortical circuitry. Dystonia can be classified according to the involved body distribution: focal, segmental, multifocal, generalized, and hemidystonia, or according to the etiology: inherited (proven genetic origin), acquired (known specific cause), and idiopathic (unknown cause) (10). Dystonia is the third most common movement disorder after PD and essential tremor (11). It is highly disabling and can seriously affect patients' quality of life. Recent studies have demonstrated that DBS of either the STN or globus pallidus internus (GPi) is an efficient treatment option for dystonia (12–16). A dual-target, crossover sham-controlled study has directly compared the clinical effects of GPi and STN stimulations in patients with dystonia, which indicated that the STN was a more effective target for dystonia than the GPi (17). STN-DBS is believed to improve the repetitive motions, abnormal posture, and quality of life of refractory patients with isolated dystonia (12, 13, 15, 18–21). The motor outcomes of STN-DBS in the treatment of dystonia have been extensively studied (12–16, 18–21). However, little is known about the effects of STN-DBS on non-motor (e.g., weight changes) symptoms in dystonia.

A thorough review of the available literature revealed that only one study has investigated WG in patients with isolated dystonia after STN-DBS, which only included nine patients (22). Given that there are significant differences in the pathophysiology and clinical manifestations of dystonia and PD (23), we investigated WG in patients with isolated dystonia who underwent STN-DBS at our center. Our center was one of the early explorers to the use of STN as a DBS target to treat isolated dystonia, which allowed us to study weight changes of a larger sample of patients with isolated dystonia after STN-DBS (24). Information on adverse events can help to better define the populations that will most benefit from STN-DBS. Thus, careful attention must be devoted to the investigation of WG after STN-DBS for the treatment of dystonia. To the best of our knowledge, this is the first study to explore the association between weight change and symptomatic improvement after STN-DBS in patients with dystonia.

## Materials and Methods

### Participants

Patients with isolated dystonia [with normal brain magnetic resonance imaging (MRI) scans] (10) who were treated with bilateral STN-DBS between August 2012 and May 2019 were included in the present study. Patients were diagnosed with isolated dystonia by movement disorder specialists and admitted to the Department of Functional Neurosurgery, Ruijin Hospital, Shanghai Jiao Tong University School of Medicine, Shanghai. All patients, in the absence of secondary causes, presented with marked symptoms, despite optimal pharmacologic treatment. Except for dystonia, all patients presented without other neurological deficiencies. The exclusion criteria were as follows: diabetes and thyroid disease, nutritional intervention received in addition to routine care, unexplained weight change, severe metabolic disease (chronic gastroenteritis, liver dysfunction, malignant tumors, other consumptive diseases, and others), severe dysphagia, history of operations (esophagectomy, bowel resection, gastrectomy, and others), active mental illness (depression, schizophrenia, and others), other brain surgery, and postoperative complications (cerebral hemorrhage, infection, and others). Patients' age ranged from 18 to 75 years. To gain insight into the etiology (idiopathic or inherited) of patients with isolated dystonia, we performed whole-exome sequencing. Thirteen patients underwent genetic testing, whereas 34 declined genetic testing. This study was approved by the ethics committee of Ruijin Hospital. Written informed consent from all patients was obtained for participation in this study.

### Data Collection

We used a structured questionnaire (32 questions) about their family and personal history, focusing on metabolic syndrome, diabetes, access to nutrition counseling, and body mass index (BMI) data. Patients with recent (<1 month) Burke–Fahn–Marsden Dystonia Rating Scale (BFMDRS) data received questionnaires via email or express delivery. The remaining patients received clinical routine outpatient follow-ups with this particular additional questionnaire. We retrospectively collected the following pre- and post-DBS data from Ruijin Hospital database of surgical patients: BFMDRS scores, weight, height, age at surgery, disease duration, and sex. Videos of BFMDRS scores were recorded by an evaluator who did not know the patient's neurostimulation status at baseline and the last follow-up postoperatively. Data from the evaluations were obtained by other evaluators (HXL and WBH) who were blinded tothe patients' names and stimulation status, using standardized scoring criteria. According to the Chinese standard “WS/T 428-2013: Criteria of weight for adults,” we classified patients into the four following groups: low weight (BMI < 18.5 kg/m^2^), normal weight (18.5 ≤ BMI < 24.0 kg/m^2^), overweight (24.0 ≤ BMI < 28.0 kg/m^2^), and obese (BMI ≥ 28.0 kg/m^2^).

### Surgical Procedure

Patients underwent standard bilateral stereotactic STN-DBS implantation procedures. MRI (1.5 T SIGNA or 3 T HDx; General Electric Company, Boston, MA, USA) and macrostimulation were used for STN targeting. Quadripolar DBS electrodes (serial number, 3387; Medtronic, Minneapolis, MN, USA or serial number, 3389; Medtronic, Minneapolis, MN, USA or L301; PINS, Beijing, China or L302; PINS, Beijing, China) were implanted under local anesthesia for precise monitoring of motor function and adverse effects using macrostimulation. An implantable pulse generator (Kinetra 7428; Medtronic or 37612; Medtronic or G101A; PINS or G102R; PINS) was implanted subclavicularly under general anesthesia. Electrode placement was validated using postoperative MRI or CT.

### Statistical Analyses

IBM SPSS Statistics for Windows version 22.0 (IBM Corp., Armonk, NY, USA) was used for data analysis. We analyzed changes in BMI, BFMDRS movement scale (BFMDRS-MS), and BFMDRS disability scale (BFMDRS-DS) using a paired Student's *t*-test or independent samples *t*-test. A one-way analysis of variance (ANOVA) was used to analyze the influence of body fat distribution on the percentage change in BMI. Multivariate linear regression was performed to evaluate the association between clinical/demographic characteristics and percentage change in BMI following surgery. The latter was regarded as dependent variables. Independent variables included sex (binary variable, where male = 0, and female = 1), age at surgery (years), preoperative BMI, time interval after surgery (months), preoperative BFMDRS-MS, and percentage change in BFMDRS-MS following surgery. All continuous data are presented as the means ± standard deviations and ranges. We report two-tailed *P*-values along with 95% confidence intervals. We selected a significance threshold of *P* = 0.05 and adjusted the significance levels for multivariate linear regression using the Benjamini–Hochberg procedure to account for multiple testing.

## Results

### Patient Characteristics

In total, 54 patients with isolated dystonia underwent bilateral STN-DBS surgery within the study period. Seven patients were excluded due to a lack of follow-up, and 47 (87%) were included in the final analysis. The results of the 13 patients who underwent genetic testing were as follows: 10 patients with idiopathic isolated dystonia, 2 patients with TOR1A (DYT1)-positive isolated dystonia, and 1 patient with THAP1 (DYT6)-positive isolated dystonia. Thirty-four patients who refused genetic testing were diagnosed with unknown (inherited or idiopathic) isolated dystonia. The body distribution of 47 patients was as follows: generalized (*N* = 14), multifocal (*N* = 17), segmental (*N* = 5), and focal (*N* = 11). These 47 patients (23 women and 24 men) were 50.62 ± 15.02 years old at the time of the operation (range, 21 to 73 years), and the average follow-up time after surgery was 39.89 ± 26.25 months (range, 4 to 83 months). The average time from diagnosis to surgery was 11.98 ± 7.68 years. The preoperative medications were as follows: 40 patients were treated with botulinum toxin, 8 with clonazepam, 17 with trihexyphenidyl, 12 with tiapride, 8 with haloperidol, 18 with baclofen, and 2 with levodopa and benserazide hydrochloride. Patient demographics and clinical data are summarized in Table 1.

**Table 1:** Demographics and dystonia- and DBS-related information.

**Demographics characteristics**	**Isolated dystonia**
No.	47
Distribution, generalized/multifocal/segmental/focal	14/17/5/11
Axis: neck and trunk/limbs: lower, upper/face: eyes and mouth/speech and swallowing	25/29/23/11
Sex, M/F	24/23
Age at surgery (years)	
Mean ± SD	50.62 ± 15.02
Range	21-73
History of disease (years)	
Mean ± SD	11.98 ± 7.68
Range	0.57-31.66
Follow-up time (years)	
Mean ± SD	3.32 ± 2.21
Range	0.36-6.96

*DBS, deep brain stimulation; SD, standard deviation*.

### Changes in Body Weight

**Figure 1:**
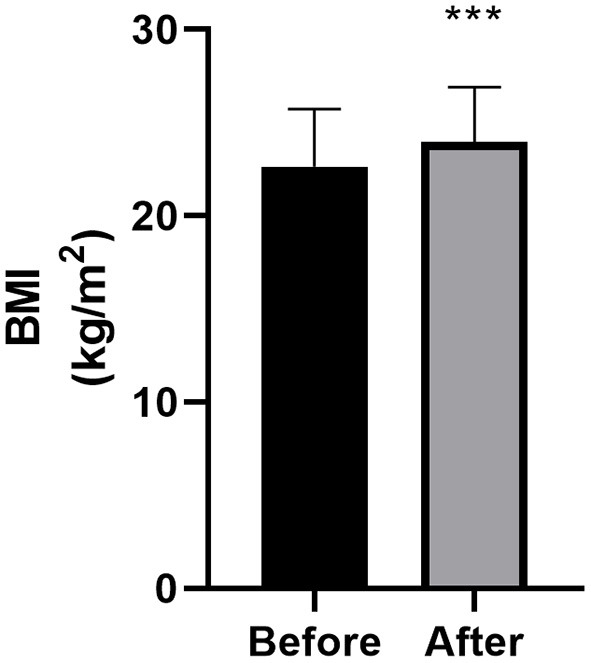
Body mass index (BMI) at baseline and final follow-up (*n* = 47). Bars represent the mean + standard deviation score. The asterisk indicates that the comparison with the preoperative score was statistically significant (***P < 0.001).

Changes in BMI are summarized in Table 2 and Figure 1. Compared with the baseline, 78.7% of the 47 patients exhibited postoperative WG at the final follow-up. The percentage of overweight or obese patients increased from 25.5 to 48.9%. The mean BMI of all patients increased by 1.32 ± 1.83 kg/m^2^ (22.64 ± 3.09 kg/m^2^ before surgery vs. 23.95 ± 2.94 kg/m^2^ after surgery; *P* < 0.001, *n* = 47), and the mean increase in BMI increase was 6.28 ± 8.34%. The mean BMI of female patients increased significantly more than that of male patients (2.02 ± 2 and 0.65 ± 1.38 kg/m^2^ for female and male, respectively; *P* = 0.009).

**Table 2:** Effect of STN stimulation on BMI at baseline and at the last follow-up in 47 patients.

**BMI groups**	**Score (kg/m**^****2****^**, mean** **±** **SD)**		***P***
	**Before surgery**	**After surgery**	
All patients (*n* = 47)	22.64 ± 3.09	23.95 ± 2.94	<0.001
Female patients (*n* = 23)	21.93 ± 2.18	23.95 ± 2.46	<0.001
Male patients (*n* = 24)	23.32 ± 3.68	23.97 ± 3.41	0.03

*STN, subthalamic nucleus; BMI, body mass index; SD, standard deviation*.

Similarly, the mean percentage change in BMI increased significantly more in female than in male patients (9.54 ± 9.32% and 3.15 ± 5.93%, respectively; *P* = 0.007). There was no significant difference in clinical characteristics between male and female patients (see Table 3). Patients were divided into three groups according to the age: <40 (*N* = 13), 40–60 (*N* = 19), and over 60 years (*N* = 15). One-way ANOVA showed no difference in percentage change in BMI among the three groups [F_2,45_ = 0.909, *P* = 0.410]. Considering the different follow-up times, patients were divided into four groups according to the follow-up time: <1 (*N* = 13), 1–3 (*N* = 8), 3–5 (*N* = 11), and more than 5 years (*N* = 16). One-way ANOVA showed no difference in the percentage change in BMI [F_3,44_ = 0.850, *P* = 0.474], BFMDRS-MS [F_3,44_ = 0.558, *P* = 0.646], and BFMDRS-DS [F_3,44_ = 0.296, *P* = 0.828] among the three groups.

**Table 3:** Clinical characteristics of male and female subjects.

**Parameters**	**Female**	**Male**	***P***
Preoperative BMI (kg/m^2^)	21.93 ± 2.18	23.32 ± 3.68	0.121
Postoperative BMI (kg/m^2^)	23.95 ± 2.46	23.97 ± 3.41	0.975
Age at surgery (years)	51.09 ± 15.63	50.17 ± 14.73	0.836
Follow-up time (years)	3.00 ± 2.35	3.63 ± 2.07	0.333
Disease duration (years)	11.63 ± 8.06	12.32 ± 7.45	0.764
Preoperative BFMDRS-MS	27.80 ± 18.66	31.13 ± 23.79	0.598
Postoperative BFMDRS-MS	8.91 ± 9.16	8.02 ± 10.68	0.760
Preoperative BFMDRS-DS	9.39 ± 6.40	11.13 ± 6.17	0.349
Postoperative BFMDRS-DS	3.13 ± 3.32	2.79 ± 2.60	0.698

*BMI, body mass index; BFMDRS, Burke–Fahn–Marsden Dystonia Rating Scale; BFMDRS-MS, BFMDRS movement score; BFMDRS-DS, Burke–Fahn–Marsden Dystonia Rating Scale disability score*.

### Changes in the Burke–Fahn–Marsden Dystonia Rating Scale

**Figure 2:**
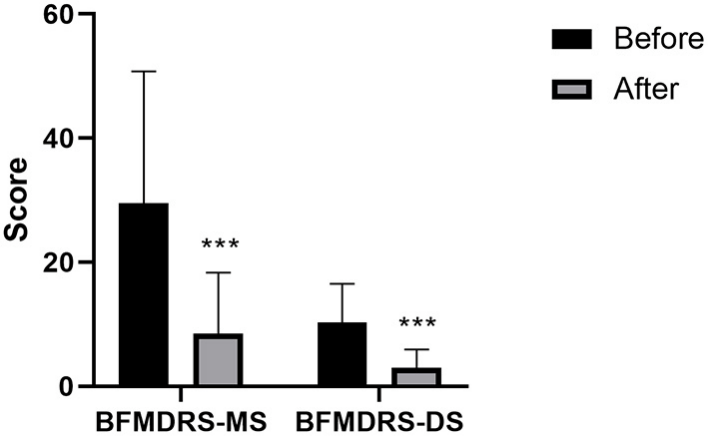
Burke–Fahn–Marsden Dystonia Rating Scale (BFMDRS) movement scores and disability scores at baseline and final follow-up (*n* = 47). Bars represent the mean + standard deviation score. Asterisks indicate where the comparisons between final follow-up score and preoperative score were statistically significant (***P < 0.001). BFMDRS-MS, BFMDRS movement score; BFMDRS-DS, BFMDRS disability score.

The changes in the BFMDRS scores are shown in Table 4 and Figure 2. Compared with those at baseline, the BFMDRS-MS and BFMDRS-DS were lower at the final follow-up. The BFMDRS-MS was reduced by 69.76 ± 33.23% (a score of 29.5 ± 21.27 before surgery vs. 8.46 ± 9.87 after surgery; *P* < 0.001, *n* = 47); for BFMDRS-DS, this was 65.66 ± 31.41% (a score of 10.28 ± 6.28 before surgery vs. 2.96 ± 2.95 after surgery; *P* < 0.001, *n* = 47).

**Table 4:** Effects of STN stimulation on BFMDRS disability scores at baseline and last follow-up in patients with isolated dystonia.

**Scale**	**Score (mean** **±** **SD)**		***P***
	**Before surgery (*n* = 47)**	**After surgery (*n* = 47)**	
BFMDRS-MS	29.5 ± 21.27	8.46 ± 9.87	<0.001
BFMDRS-DS	10.28 ± 6.28	2.96 ± 2.95	<0.001

*SD, standard deviation; BFMDRS, Burke–Fahn–Marsden Dystonia Rating Scale; BFMDRS-MS, BFMDRS movement score; BFMDRS-DS, BFMDRS disability score*.

### Factors Associated With Body Mass Index Gain

In the univariate linear regression analysis, age at surgery, follow-up period, baseline BFMDRS-MS, and percentage change in BFMDRS-MS were not associated with percentage change in BMI (*P* > 0.05; see Table 5); thus, we only added sex and preoperative BMI to the multivariate linear regression model. These were both independently associated with an increase in BMI (*P* < 0.05; see Table 6).

**Table 5:** Clinical and demographic predictors of changes in BMI after surgery.

**Parameters**	**Coefficient**	**Standard error**	**Beta**	***t***	***P***
Sex (female)	6.389	2.259	0.387	2.817	0.007^*^
Age at surgery (years)	−0.072	0.082	−0.129	−0.871	0.388
Pre-surgical BMI	−1.104	0.367	−0.409	−3.009	0.004^*^
Disease duration (years)	0.167	0.160	0.154	1.042	0.303
Time interval after surgery (years)	0.756	0.551	0.200	1.372	0.177
Pre-surgical BFMDRS-MS	0.015	0.058	0.039	0.261	0.795
Percent change in BFMDRS-MS scores (%)	−0.014	0.037	−0.058	−0.386	0.701

*BMI, body mass index; BFMDRS-MS, Burke–Fahn–Marsden Dystonia Rating Scale movement score*.

* *P < 0.05*.

**Table 6:** Clinical and demographic predictors of changes in BMI after surgery.

	**Coefficient**	**Standard error**	**Beta**	***t***	***P***
Sex	5.115	2.200	0.310	2.325	0.025^*^
Pre-surgical BMI	−0.913	0.360	−0.339	−2.540	0.015^*^

*BMI, body mass index*.

* *P < 0.05*.

According to the distribution of dystonia, the 47 patients with dystonia were divided into four groups, as follows: focal (*n* = 11), segmental (*n* = 5), multifocal (*n* = 17), and generalized (with or without eyelid involvement) (*n* = 14). The one-way ANOVA indicated no difference in the percentage change of BMI before and after operation [F_3, 43_ = 0.465, *P* = 0.708] or BFMDRS-MS [F_3,43_ = 0.484, *P* = 0.695]. Patients were divided into two groups according to their dyskinesias, as follows: group 1, with limb involvement (*n* = 30); and group 2, without limb involvement (*n* = 17). The results of the independent samples *t*-test revealed no difference in the percentage change in BMI (*P* = 0.346) or BFMDRS-MS (*P* = 0.477) between the two groups.

Five patients in whom only the eyelids were affected were regarded as one group, and the other patients (with or without eyelid involvement) (*n* = 42) were regarded as another group. The results of an independent samples *t*-test revealed no difference in the percentage change in BMI (*P* = 0.862) or BFMDRS-MS (*P* = 0.499) between the two groups.

## Discussion

In this study, we confirmed that patients with isolated dystonia typically exhibit WG after STN-DBS. Overall, 78.7% of the patients exhibited postoperative WG at the final follow-up. The proportion of patients that were overweight or obese increased from 25.5% before surgery to 48.9% in the final follow-up period.

There was a significant correlation between WG and sex, whereby the mean BMI of female patients increased significantly more than that of male patients after surgery. Considering that the average age of female patients at surgery was 51 years, this association may be associated with changes in menopausal hormones (25). This correlation has been previously identified for patients with PD who underwent STN-DBS (26). There is evidence showing that female patients with PD gain disproportionate amounts of fat mass after STN-DBS, while WG in men was driven by both fat-free mass and fat mass (27, 28). In this study, the percentage of overweight or obese female patients increased by 34.8% (8.7% before surgery vs. 43.5% after surgery), while that of men increased by 12% (42% before vs. 54% after surgery). In this context, it is also important to assess the changes in body composition after STN-DBS.

We found that the average percentage reduction in BFMDRS-MS was 69.76 ± 33.23%. However, the baseline BFMDRS-MS and percentage change in BFMDRS-MS were not associated with WG. There are three possible explanations for this result. First, BFMDRS-MS evaluates not only the limbs and trunk but also the eyes, the mouth, speech, swallowing, and the neck; thus, the affected body part may not be associated with resting EE (REE). There were only three patients with low weight before the operation, which may also indicate that the slight movement disorder before the operation had little effect on the patients' REE. Second, patients were able to perform larger movements and were more mobile after the operation than before the operation, which should theoretically increase EE. This may offset the reduced EE caused by improved motor symptoms. Of course, since we did not evaluate the free-living EE in a calorimetric chamber, the decrease in total daily EE due to the reduction in dyskinesias is uncertain. Third, we regrouped the patients three times (according to the distribution of the dystonia, dyskinesias, and involvement of the eyelids) and analyzed the subgroups separately. There were no differences in the percentage change of postoperative BMI and BFMDRS-MS between subgroups. Initially, we assumed that there would be no difference in postoperative physical activity in patients with involvement of only the eyelids before and after surgery and that there would be no difference in REE or free-living EE before and after surgery in this subgroup. However, the BMI of these patients differed before and after surgery. Therefore, the observed WG may result from changes in the central regulation of energy metabolism caused by STN-DBS. Concerning WG after STN-DBS in patients with PD, various studies have discovered no correlation between WG and changes in the Unified Parkinson Disease Rating Scale III score (28, 29). For future studies, it will be important to measure REE and free-living EE. Moreover, the reduction in EE may be due to the reduction in sustained or intermittent muscle contractions; however, changes in the central regulation of energy metabolism caused by direct STN-DBS may also be involved.

Indeed, previous studies have illustrated that a change in body weight is the direct result of STN-DBS on the regulation of the dietary and metabolic nucleus (in the lateral region of the thalamus and the marginal portion of the STN) (30–34). STN-DBS may have a regional effect on the hypothalamic–pituitary–adrenal homeostatic control of EE by diffusion of the electrical pulse to the hypothalamus (35, 36). Results of the direct effects of STN-DBS on hypothalamic catabolic/anabolic regulators including blood cortisol (36), ghrelin, neuropeptide Y, and leptin peptides (37–39) remain inconsistent and require further study. Therefore, the improvement of motor symptoms in dystonia is insufficient to explain WG after STN-DBS.

The balance between energy intake and EE determines weight change. However, there are no data on the difference in pre- and postoperative energy intake for STD-DBS in patients with isolated dystonia. Notably, a number of researchers have observed that WG after STN-DBS in patients with PD could result from increased sensitivity to food reward cues (40) and changes in eating behavior, including higher food intake, increased appetite, binge eating, or cravings (41–45). However, there have also been reports of no significant changes in the quality or quantity of daily food intake after surgery (41, 46). These inconsistencies could be explained by inaccuracies in food intake measurements. Patients with PD display a large amount of interindividual variation of non-motor symptoms including autonomic dysfunction, olfactory hypoxia, mood disturbances, sleep disorders, and gastrointestinal dysfunction. However, the association of non-motor symptoms with body weight and daily food intake in patients with PD has not been confirmed, and findings have often been contradictory (47, 48). Patients with dystonia also show non-motor symptoms including pain, cognitive, and sleep disturbances (8); therefore, the association between non-motor symptoms and weight change in patients with dystonia deserves further study.

The limitations of the current study include its retrospective study design and the absence of a control group. As this was a retrospective study, relevant preoperative data (hormonal and dietary diaries represent) are not available. We did not prospectively collect the patients' movement scores and BMI information during multiple follow-ups. Despite the follow-up time in our study, ranging from 4 to 83 months, previous studies have revealed that patients with isolated dystonia can achieve near-maximum improvement in motor scores and remain relatively stable for 3 months after STN-DBS (13). Despite a wide age range and different follow-up times, the one-way ANOVA showed no difference in the percentage change in BMI, BFMDRS-MS, and BFMDRS-DS in different subgroups. Nevertheless, the conclusions that can be drawn from this study are limited by the heterogeneity of isolated dystonia, age range of patients, and follow-up time. In the future, a larger sample size is required to study weight change after STN-DBS in patients with isolated dystonia, as well as to explore the mechanisms underlying WG after treatment. Also, due to the heterogeneity of isolated dystonia and the relatively small number of dystonia patients, we do not have data on the GPI-DBS matching STN-DBS group. Therefore, we did not use the GPI-DBS treatment group as the control group. Interestingly, the WG in patients with PD after STN-DBS was more significant than that in patients with PD after GPI-DBS (22).

Large-scale systematic studies are required to determine the reasons for postoperative weight change. Future studies should also focus on changes in non-motor symptoms, food intake, and EE (resting/free-living) after STN-DBS in patients with isolated dystonia. In addition, those patients in whom sustained or intermittent muscle contractions has little to no effect on the range of physical activity (such as those with dystonia involving only the eyelids) warrant further study. Such patients may be better disease models for studying weight change after STN-DBS. Their postoperative REE and free-living EE should be similar before and after STN-DBS, which should help elucidate reasons (such as changes in energy metabolism caused by direct stimulation), other than the improvement of motor symptoms, that lead to postprocedural weight change.

## Conclusions

To our knowledge, this is the first report to investigate changes in body weight after STN-DBS in patients with isolated dystonia. We found that STN-DBS is an effective treatment to relieve symptoms of isolated dystonia, but that it may cause postoperative WG, especially in female patients. Such WG was correlated with preoperative weight, but not to DBS treatment efficacy of, for example, motor symptoms. Clinical teams must be more careful in choosing STN as a therapeutic target for DBS in patients with dystonia who are overweight or obese, especially women. Although this is the first case investigating change in body weight after STN-DBS in patients with isolated dystonia, more studies and long-term follow-ups are needed to determine the mechanisms underlying WG after STN-DBS operation.

## Data Availability Statement

The original contributions presented in the study are included in the article/supplementary material, further inquiries can be directed to the corresponding author/s.

## Ethics Statement

The studies involving human participants were reviewed and approved by the ethical committee of Ruijin Hospital. The patients/participants provided their written informed consent to participate in this study.

## Author Contributions

WH: research project execution, statistical analysis design and execution, and manuscript writing of the first draft. HL: research project execution, and statistical analysis execution. YL and YuW: statistical analysis design and execution. YiW: research project conception and organization, statistical analysis design, review, critique, and manuscript review and critique. AR-Z: research project conception and organization, statistical analysis design, review, critique, and manuscript review and critique. WY and CZ: research project conception and organization, statistical analysis design, review, critique, and manuscript review and critique. All authors contributed to the article and approved the submitted version.

## Conflict of Interest

The authors declare that the research was conducted in the absence of any commercial or financial relationships that could be construed as a potential conflict of interest.
